# The level of lipopolysaccharide-binding protein is elevated in adult patients with obstructive sleep apnea

**DOI:** 10.1186/s12890-018-0647-z

**Published:** 2018-05-29

**Authors:** Yinfeng Kong, Zhijun Li, Tingyu Tang, Haiyan Wu, Juan Liu, Liang Gu, Tian Zhao, Qingdong Huang

**Affiliations:** Department of Respiratory Medicine in Zhejiang Hospital, 12 Lingyin Road, Xihu District, Hangzhou, 310013 Zhejiang Province China

**Keywords:** Obstructive sleep apnea, Lipopolysaccharide-binding protein, Inflammation, Serum

## Abstract

**Background:**

lipopolysaccharide-binding protein (LBP) has been to be a surrogate marker of inflammation in OSA. This study aimed to test the hypothesis that the concentration of LBP is elevated in adult patients with obstructive sleep apnea (OSA).

**Methods:**

A total of 90 patients were enrolled into the study, 50 subjects were divided into OSA groups and 40 in healthy control according to PSG examination. Subsequently, patients with apnea-hypopnea index (AHI) ≧ 5, were divided into different subgroups according to blood pressure, gender, body mass index (BMI) and AHI. Venous blood samples were collected for detection after polysomnography. The serum levels of LBP and proinflammatory cytokines (interleukin (IL)-1β, IL-6, tumor necrosis factor (TNF)-α) were tested by ELISA.

**Results:**

The present study demonstrated that the serum levels of both LBP and proinflammatory cytokines were elevated in OSA patients. A stratified analysis conducted to analyze differences among subgroups indicated that OSA patients with a higher AHI or BMI had an increased level of LBP and proinflammatory cytokines (all *p* < 0.05). Furthermore, a significant correlations were observed between LBP and inflammation and AHI. Multivariate regression analysis also demonstrated that AHI, LSaO2 and BMI had impact on the concentration of LBP.

**Conclusion:**

The research showed that the serum level of LBP and proinflammatory cytokines were elevated in adult patients with OSA, and an association with severity of disease and BMI were established. Furthermore, sleep apnea and BMI had effect on the concentration of LBP.

## Background

Obstructive sleep apnea (OSA) is described as repeated collapse of the upper airways during sleep, leading to repeated cycle of hypoxemia-reoxygenation and sleep disruption. Risk factors for OSA, including obesity and aging, are on the rise in the public; therefore, the prevalence of OSA is increasing worldwide, and is estimated to affect up to 17% of middle-aged men and 9% of middle-aged women [1].

The putative mechanism by which OSA has been linked to numerous pathologic conditions including stroke, cardiovascular disease, hypertension, and metabolic derangements is through the systemic inflammatory cascade [2–4]. In the past several years, it has become apparent that the increasing level of inflammation are strongly associated with OSA [5]. A meta-analysis including 1985 OSA patients and conducted by Xie et al. indicated that proinflammatory factors, such as interleukin (IL)-6, IL-8, and tumor necrosis factor (TNF)-α, are increased in patients with OSA, which is partially reversed after continuous positive airway pressure intervention [6]. However, the potential molecular mechanisms how to initiate the inflammation are not fully understood in OSA patients.

Lipopolysaccharide-binding protein (LBP) is an acute-phase reactant predominantly derived from the liver, adipose and intestinal epithelial cells. LBP binds lipopolysaccharide (LPS) through recognition of lipid A and initiates its response by forming a complex with myeloid differentiation factor 2 leading to activation of both MyD88-dependent and non-MyD88-dependent downstream signaling pathways causing subsequent inflammatory responses, such as the release of various biomediators including IL-6, TNF-α, and IL-1β [7]. Therefore, LBP usually as a biomarker of system inflammation, intestinal barrier and microbe translocation in deferent study [8–11].

Metabolic endotoxemia has been shown to be the primary contributor to the pathogenesis of chronic low-grade inflammation, characterized by increased plasma LBP levels, which are believed to originate from changes in gut microbiota and increasing of intestinal permeability [10]. Altered gut microbiota composition are key factors affecting gut barrier integrity. The gut microbiota, which serves as reservoir for bacterial LPS, could be altered by OSA and subsequently trigger inflammation. At present, several studies reported that the chronic intermittent hypoxia of OSA has a significant impact on the overall microbial community structure of mice, indicating that the homeostatic relationships between host and gut microbiota could be compromised in OSA patients [12, 13]. Moreno-Indias et al. study indicated that fecal microbiota composition and diversity were altered as a result of intermittent hypoxia realistically mimicking OSA [12]. Therefore, it is rational to deduce that the concentration of LBP is elevated in OSA patients. At present, one study from Kheirandish-Gozal et al. reported that children with OSA exhibited increased LBP levels [14]. However, only children were recruited into the Kheirandish-Gozal et al. research. There is indeed a paucity of published literature on the association between LBP levels and adult patients with OSA.

Based on previous studies, we conducted the study to test the hypothesis that the serum level of LBP is elevated in adult patients with OSA, and the correlations with proinflammatory factors and AHI were also evaluated.

## Methods

### Study population

A total of 50 patients with OSA (mild [*n* = 10], moderate [*n* = 15], and severe OSA [*n* = 25]) and 40 healthy controls were consecutively recruited in the study. The patients were examined at sleep laboratory of the respiratory department of Zhejiang Hospital from January 2016 to June 2016. Body weight and height were measured, and body mass index (BMI) was calculated as weight (kg)/height^2^(m), and overweight was defined as BMI ≥ 25 kg/m^2^. Blood pressure was recorded using a mercury sphygmomanometer, and elevated blood pressure was defined as systolic blood pressure (SBP) ≧ 140 mmHg and/or diastolic blood pressure (DBP) ≧ 90 mmHg or taking antihypertensive drug.

All subjects provided their informed written consent. The study was conducted according to the World Medical Association Declaration of Helsinki in 1975, as revised in 1983, and was approved by the Ethic Committee of Zhejiang Hospital.

### Polysomnography

All participants received overnight polysomnography (PSG) according to standardized criteria [15]. The results of PSG were reviewed by two sleep specialists (Juan Liu & Liang Gu). Apnea was defined as continuous cessation of airflow for > 10 s. Hypopnea was defined as a ≥ 30% reduction in airflow for > 10 s with oxygen desaturation of ≥4%. Apnea-hypopnea index (AHI) was calculated as the sum of apneas and hypopneas per hour during overnight. A respiratory event was scored as an obstructive apnea or hypopnea if chest and abdominal respiratory movement was identified and oronasal airflow ceased, or there is an associated thoracoabdominal paradox that occurs during the hypopnea with snoring. Microarousal index (MAI) was defined according to the American Academy of Sleep Medicine Scoring Manual [16]. The oxygen desaturation index (ODI) was defined as ≥4% oxygen desaturation per hour during sleep. Patients with OSA were divided into the mild group (AHI: 5–15 events/h), moderate group (AHI: 15–30 events/h), and severe group (AHI > 30 events/h) [17]. Subjects with sleepy and snore who accepted PSG examination, with an AHI < 5 events/h were included in the study as healthy controls. The exclusion criteria of subjects were as follows: (1) chronic hypoxia caused by asthma, chronic obstructive pulmonary disease, interstitial lung disease and other respiratory disorders. (2) Participants with a history of drug or alcohol abuse, or taking drugs to regulate intestinal flora. (3) cardiovascular, endocrine, and other disorders that could lead to hypoxemia. (4) Diseases which may lead to the release of proinflammatory factors, such as connective tissue disease, cancer, and inflammatory bowel disease. (5) required gastrointestinal surgical procedures or had received antibiotic therapy in the preceding 8 weeks were also excluded.

### Blood collection and analysis

Peripheral blood, drawn from each subject on the morning after PSG, was centrifuged at 3000 rpm for 15 min, and serum was stored at − 70 °C for analysis. Concentrations of serum LBP, IL-1β, IL-6, TNF-ɑ were measured using commercially available ELISA kits (R&D Systems, Minneapolis, MN, USA) in duplicate according to the manufacturer’s instruction. An automatic biochemical analyzer (UniCel DxC 800 Synchron, Beckman Coulter, Inc., Brea, CA, USA) was used to test the serum level of lipids.

### Statistical analysis

Continuous variables are expressed as the mean ± standard deviation. Comparisons were performed using *t* test or χ^2^ tests depending on data differences among groups. Spearman’s correlation analysis were conducted to examine potential associations between LBP and proinflammatory factors. A multivariate regression analysis was also performed to evaluate the role of confounding factors on LBP. Statistical significance was determined by a level of 0.05 on two-sided tests. All statistical analysis was performed using the SPSS Statistics 19.0.0 (IBM Corporation, Somers, NY, USA).

## Results

### General clinical characteristics of the study participants

The basic clinical characteristics of the subjects are detailed in Table 1. The mean age of patients with OSA was 54.34 ± 14.38 years, compared with 50.42 ± 8.35 years in the control group. There are significant differences were observed between cases and controls group in terms of BMI, blood pressure (SBP and DBP), the serum concentration of triglyceride (TG), and PSG parameters (all *p* < 0.05).

**Table 1:** Clinical characteristics of the OSA and control group

Variables	Patients	Controls	*p* value
Gender			0.317
Male	34	31	
Female	16	9	
Age (years)	54.34 ± 14.38	50.42 ± 8.35	0.112
BMI(kg/m^2^)	26.86 ± 3.12	22.26 ± 3.54	0.000
Normal weight	11(22.00%)	27(67.50%)	
Overweight	39(78.00%)	13(32.50%)	
SBP(mmHg)	133.60 ± 16.37	120.19 ± 17.11	0.000
DBP(mmHg)	81.16 ± 14.45	74.05 ± 10.72	0.005
Respiratory events			
Obstructive	241.76 ± 106.12	17.49 ± 7.18	< 0.001
Central	1.32 ± 0.59	0.94 ± 0.37	0.551
AHI	37.34 ± 19.02	3.31 ± 1.09	< 0.001
LSaO2	75.29 ± 11.83	95.95 ± 4.65	< 0.001
mSaO2	93.01 ± 4.18	96.35 ± 3.87	< 0.001
ODI	41.83 ± 25.9	3.56 ± 1.12	< 0.001
MAI	22.61 ± 15.12	4.27 ± 2.16	< 0.001
NREM1(%)	23.21 ± 12.79	15.9 ± 13.04	0.031
NREM21(%)	52.73 ± 12.31	48.1 ± 16.21	0.335
NREM31(%)	14.51 ± 9.95	20.23 ± 8.72	0.045
REM(%)	10.23 ± 5.31	16.74 ± 7.36	0.012
TG	2.96 ± 2.53	1.71 ± 1.07	0.004
TC	4.98 ± 1.10	4.71 ± 0.91	0.189
HDL	1.04 ± 0.23	1.23 ± 0.26	0.000
LDL	2.98 ± 1.10	3.02 ± 0.89	0.847
LBP	36.05 ± 7.35	32.11 ± 5.94	0.010
IL-1β	27.15 ± 5.91	21.17 ± 1.70	0.000
IL-6	61.59 ± 9.76	54.46 ± 9.43	0.001
TNF-ɑ	327.34 ± 46.81	307.95 ± 27.15	0.027

*AHI* Apnea-hypopnea index, *BMI* Body mass index, *SBP* Systolic blood pressure, *DBP* Diastolic blood pressure, *LSaO2* Lowest saturation oxygen, *mSaO2* Mean saturation oxygen, *ODI* Oxygen desaturation index, *MAI* Microarousal index, *TG* triglyceride, *TC* Total cholesterol, *LDL* Low-density lipoprotein, *HDL* High-density lipoprotein

### The comparison of LBP and proinflammatory factors between patients and controls

There were significant differences in the serum level of LBP (36.05 ± 7.35 vs 32.11 ± 5.94, *p* = 0.01) and inflammatory factors (IL-1β, 27.15 ± 5.91 vs 21.17 ± 1.70, *p* = 0.000; IL-6, 61.59 ± 9.76 vs 54.46 ± 9.43, *p* = 0.005; TNF-ɑ 327.34 ± 46.81 vs 307.95 ± 27.15, *p* = 0.020) between cases and control group.

To further analyze differences among subgroups, a stratified analysis was performed according to blood pressure, gender, BMI and severity of disease (AHI). The results demonstrated that a significant differences were found between normal weight and overweight patients with OSA, which suggested that OSA patients with a higher BMI had a higher serum level of LBP and proinflammatory factors (all *p* < 0.05). However, no differences were identified in other subgroups based on blood pressure and gender (*p* > 0.05) (Table 2). Alternatively, a marked differences were determined in the mild vs moderate, mild vs severe, moderate vs severe groups (all *p* < 0.05), except for IL-1β in the mild vs moderate group (*t* = − 1.837, *p* = 0.091) (Table 3).

**Table 2:** Comparison of various index in OSA subgroups according to blood pressure, gender and BMI

Variables	HBP vs Normal BP		*p* value	Male vs Female		*p* value	Normal weight vs Overweight		*p* value
	HBP(21)	Normal BP(29)		Male(34)	Female(16)		Normal weight(14)	Overweight(36)	
Age(years)	49.76 ± 13.28	56.46 ± 14.55	0.083	55.76 ± 12.76	52.13 ± 9.41	0.912	58.36 ± 18.68	52.78 ± 12.29	0.315
BMI(kg/m^2^)	28.30 ± 3.32	25.09 ± 2.83	0.022	27.28 ± 2.79	25.44 ± 3.12	0.531	24.42 ± 1.17	27.56 ± 2.60	0.017
SBP(mmHg)150.06 ± 7.61	123.61 ± 11.33	0.000	135.12 ± 15.61	131.50 ± 17.14	0.362	126.15 ± 14.31	136.63 ± 16.38	0.051	
DBP(mmHg) 92.71 ± 13.12	74.14 ± 10.15	0.000	83.08 ± 14.23	78.42 ± 9.12	0.112	75.85 ± 11.83	83.31 ± 15.02	0.117	
AHI	34.43 ± 17.72	40.19 ± 20.51	0.322	40.35 ± 16.63	29.61 ± 23.02	0.073	33.87 ± 16.52	38.69 ± 19.96	0.426
LSaO2(%)	77.17 ± 10.71	71.83 ± 13.75	0.160	72.87 ± 12.24	75.85 ± 7.77	0.132	75.83 ± 11.09	75.12 ± 12.22	0.859
mSaO2(%)	92.96 ± 5.37	93.67 ± 2.30	0.684	92.73 ± 4.78	94.11 ± 1.54	0.315	93.74 ± 2.67	92.89 ± 4.59	0.546
ODI	36.58 ± 23.77	48.89 ± 27.72	0.126	47.75 ± 24.49	25.91 ± 23.50	0.008	36.71 ± 22.46	43.54 ± 27.03	0.435
MAI	20.13 ± 13.27	26.33 ± 17.25	0.180	24.49 ± 16.22	17.44 ± 10.45	0.170	20.25 ± 13.07	23.56 ± 15.97	0.512
LBP	36.25 ± 8.71	35.72 ± 4.52	0.805	35.78 ± 7.73	37.14 ± 5.93	0.645	26.75 ± 3.50	37.38 ± 6.79	0.002
IL-1β	26.89 ± 6.27	27.58 ± 5.44	0.728	27.81 ± 5.93	24.52 ± 5.36	0.161	20.90 ± 2.03	28.04 ± 5.75	0.010
IL-6	61.69 ± 10.62	61.46 ± 8.47	0.944	61.52 ± 10.22	61.90 ± 8.24	0.924	46.41 ± 5.50	63.78 ± 8.18	0.000
TNF-α	325.11 ± 49.82	331.06 ± 42.73	0.703	329.95 ± 49.06	316.88 ± 37.36	0.487	252.99 ± 18.74	337.96 ± 39.29	0.000

*AHI* Apnea-hypopnea index, *BMI* Body mass index, *SBP* Systolic blood pressure, *DBP* Diastolic blood pressure, *LSaO*_*2*_ Lowest saturation oxygen, *mSaO*_*2*_ Mean saturation oxygen, *ODI* Oxygen desaturation index, *MAI* Microarousal index

**Table 3:** Comparison of various index in subgroups according to severity of disease

Variables	OSA			Mild vs Moderate		Mild vs Severe		Moderate vs Severe	
	Mild(10)	Moderate(15)	Severe(25)	t	*p* value	t	*p* value	t	*p* value
Age(years)	59.60 ± 15.13	56.73 ± 15.93	52.27 ± 13.54	0.352	0.729	1.104	0.277	−0.983	0.331
BMI(kg/m^2^)	27.04 ± 2.58	26.81 ± 3.80	27.28 ± 3.16	0.120	0.906	−0.164	0.871	0.436	0.665
SBP(mmHg) 140.40 ± 13.05	130.31 ± 16.79	133.93 ± 16.82	1.204	0.246	0.812	0.423	0.638	0.528	
DBP(mmHg)	85.20 ± 9.61	79.85 ± 15.63	81.04 ± 14.63	0.686	0.502	0.597	0.555	0.236	0.815
AHI	10.14 ± 2.95	22.88 ± 3.93	49.11 ± 14.96	−6.066	0.000	−12.848	0.000	−8.999	0.000
LSaO2	87.20 ± 1.30	79.53 ± 9.13	70.90 ± 12.00	3.159	0.006	6.958	0.000	2.429	0.020
mSaO2	94.80 ± 1.48	93.88 ± 2.27	92.38 ± 5.10	0.840	0.412	1.040	0.307	1.077	0.288
ODI	11.94 ± 5.37	26.41 ± 18.78	55.44 ± 22.17	−1.672	0.112	−9.008	0.000	−4.305	0.000
MAI	14.56 ± 2.87	16.45 ± 8.12	27.06 ± 17.40	−0.501	0.623	−3.484	0.002	−2.627	0.012
LBP	25.81 ± 7.16	33.55 ± 3.50	38.29 ± 7.40	−2.743	0.018	− 2.771	0.010	− 2.642	0.012
IL-1β	20.10 ± 1.47	24.35 ± 3.83	29.15 ± 5.91	−1.837	0.091	−2.604	0.015	−2.475	0.018
IL-6	46.20 ± 6.09	58.34 ± 7.09	64.76 ± 9.05	−2.686	0.020	−3.434	0.002	− 2090	0.044
TNF-α	274.64 ± 4.91	308.75 ± 42.48	341.28 ± 44.80	−2.601	0.025	−2.534	0.017	−2.048	0.048

*AHI* Apnea-hypopnea index, *BMI* Body mass index, *SBP* Systolic blood pressure, *DBP* Diastolic blood pressure, *LSaO*_*2*_ Lowest saturation oxygen, *mSaO*_*2*_ Mean saturation oxygen, *ODI* Oxygen desaturation index, *MAI* Microarousal index

### The correlations between LBP and proinflammatory factors and severity of disease

**Fig. 1:**
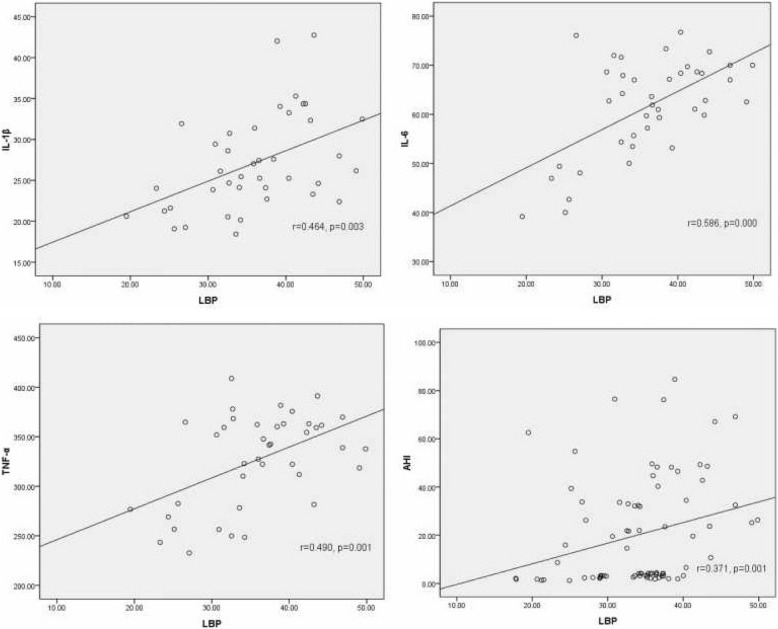
The scotterplots of the correlation of LBP vs IL-1β, IL-6, TNF-α and AHI

Based on aforementioned findings, The correlations between LBP and proinflammatory cytokines and severity of diseases were also evaluated. As evident in Fig. 1, significant correlations were found between LBP and IL-1β (*r* = 0.464, *p* = 0.003), IL-6 (*r* = 0.586, *p* = 0.000), TNF-ɑ (*r* = 0.490, *p* = 0.001), and AHI (*r* = 0.371, *p* = 0.001). In addition, a multivariate regression analysis were performed to determine the role of possible confounding factor to the concentration of LBP. The result of analysis showed that AHI, LSaO2 and BMI had a significant impact on the LBP (all *p* < 0.05), which suggested that sleep apnea and obesity have effect on the levels of serum LBP. However, other variables were not identified (*p* > 0.05) (Table 4).

**Table 4:** The effect of confounding factor on concentation of LBP in OSA patients

Variables	*β*	95% CI	*p* value
AHI	14.430	(5.152, 29.078)	0.026
ODI	−0.139	(−0.397, 0.119)	0.276
MAI	0.074	(−0.135, 0.282)	0.472
LSaO2	10.125	(3.917, 25.071)	0.035
mSaO2	−0.256	(− 0.929, 0.416)	0.438
Age	0.115	(−0.150, 0.379)	0.379
BMI	8.245	(1.154, 18.386)	0.041
SBP	−0.214	(− 0.470, 0.042)	0.097
DBP	0.345	(−0.008, 0.698)	0.055
TG	1.378	(−0.964, 3.719)	0.236
TC	−5.532	(−12.988, 1.924)	0.138
HDL	−4.509	(−20.564, 11.546)	0.567
LDL	5.397	(−2.166, 12.960)	0.153
IL-1β	0.373	(−0.730, 1.476)	0.492
IL-6	−0.079	(−0.363, 0.204)	0.569
TNF-α	−0.006	(−0.076, 0.064)	0.859

*AHI* Apnea-hypopnea index, *BMI* Body mass index, *SBP* Systolic blood pressure, *DBP* Diastolic blood pressure, *LSaO*_*2*_ Lowest saturation oxygen, *mSaO*_*2*_ Mean saturation oxygen, *ODI* Oxygen desaturation index, *MAI* Microarousal index

## Discussion

This is the first study to investigate OSA has an impact on the concentration of LBP in adults. The results demonstrated that the serum level of LBP and inflammation were higher in OSA patients, compared with healthy subjects, and subgroups analysis indicated that OSA patients with a higher BMI and AHI had a higher serum level of LBP and proinflammatory factors. Additionally, the correlational analysis showed that serum LBP levels were positively correlated with inflammation and AHI. A multivariate regression analysis indicated that sleep apnea and BMI had a significant impact on the concentration of LBP.

To the best of our knowledge, OSA has an impact on the intestinal barrier function and gut microflora. A study conducted by Moreno-Indias et al. suggested that composition and diversity of intestinal microflora are altered caused by intermittent hypoxia [12]. The translocation or change of commensal microbiota across the intestinal barrier can result in a persistent state of low grade immune activation or inflammation. LBP is located upstream of IL-1β, IL-6, and TNF-α expression, and initiates the recognition of bacterial LPS exposure and amplifies the host immune response which, if continued long-term, results in adverse sequelae to the host. Therefore, LBP has been suggested to serve as a surrogate marker of chronic inflammatory status in several disorders, such as obesity, diabetes, hypertension and other chronic inflammation diseases [18, 19]. Kim et al. study indicated that LBP levels were positively associated with BMI, SDP, total cholesterol, low density lipoprotein-cholesterol, fasting glucose and insulin, and insulin resistance [18].

The present study showed that the levels of LBP and inflammation are increased in adult OSA patients and are positively associated with the severity of disease. Significant correlations were also determined between LBP and AHI and proinflammatory factors, which was is in line with the previous study. Another study of Moreno-Indias et al. demonstrated that the LPS concentration was elevated at the end of intermittent hypoxia in mouse model, and a significant association was found between gut bacterial dysbiosis and the increases in plasma LPS levels [20]. In a recent study conducted by Kheirandish-Gozal et al. [14], they assessed the LBP plasma levels of 219 child patients with OSA, and found that systemic low-level endotoxemia and elevation of LBP was established in children with OSA, associated with measures of OSA severity. However, only children with OSA were included to analyze in the study. Another research conducted by Sakura et al. also domenstrated that serum LBP levels were positively correlated with inflammation [19]. Taken together, we can deduce that chronic intermittent hypoxia of OSA may lead to elevation of systemic LBP levels with resultant inflammation by causing disorder of intestinal microflora.

As previously described, several researches reported that LBP levels and proinflammation factors had positively associated with BMI and hypertension [18]. Although a remarkable differences were obsevered between cases and controls in term of BMI and blood pressure. However, the result of present study demonstrated that the concentration of LBP and level of inflammation had only a positive association with BMI in OSA patients, and no relation with hypertension. As we known, obesity is one of the strongest risk factors for OSA, which imposes mechanical loads on the upper airway, resulting in flow limitation and apnea, with > 50% of OSA diagnoses attributable to being overweight [21]. Kheirandish-Gozal et al. also indicated that a significant increases was identified in LBP levels in children with obesity or OSA, and the highest LBP levels was observed when both conditions are present [14]. Additionally, a study came from Kim et al. showed that circulating plasma LBP levels were significantly increased in overweight/obese participants compared with those in normal weight participants [18]. The same results drew from another study conducted by Gonzalez-Quintela et al. [22]. Therefore, the current evidence supported that that sleep apnea and other factors such as obesity may disrupt intestinal barrier function or gut microbiota, and casue to increased the serum LPS concentration with resultant systemic inflammation.

In interpreting the results of the research, there are some methodological limitations requiring comment. First, disruption of gut microflora plays an important role in low-grade inflammation, we should therefore detect changes of gut microflora in future studies to explain the underlying mechanism of increased of LBP and inflammatory factors caused by OSA. Second, the study was not a clinical randomized controlled trial, and only the serum level of LBP and proinflammatory factors were evaluated in patients with OSA. It would be important to test if we can detect the changes in LBP and proinflammatory factors levels before and after intervention. Third, the sample size of the study was relatively small, with only 50 OSA patients included. These limitations could have affected the power of the conclusions. In the future, we intend to conduct an analysis of a larger sample size and a randomized controlled trial, which will include these risk factors.

## Conclusions

The present study shown that higher LBP levels and inflammation are detected in the presence of obesity and in the presence of sleep-disordered breathing in a severity-dependent fashion. Furthermore, higher serum LBP levels were positively correlated with AHI, and sleep apnea and BMI had effect on the concention of LBP. Improved understanding the mechanism underlying these associations may offer not only opportunities for detection of OSA patients at risk of comorbidities, but may also enable delineation of therapeutic interventions, such as regulation of intestinal flora through probiotics, for example, to reduce end-organ damage caused by OSA.

## Abbreviations

AHI: Apnea-hypopnea index
BMI: Body mass index
DBP: Diastolic blood pressure
HDL: High-density lipoprotein
IL: Interleukin
LBP: Lipopolysaccharide-binding protein
LDL: Low-density lipoprotein
LPS: Lipopolysaccharide
LSaO2: Lowest saturation oxygen
MAI: Microarousal index
mSaO2: Mean saturation oxygen
ODI: Oxygen desaturation index
SBP: Systolic blood pressure
TC: Total cholesterol
TG: Triglyceride
TNF: Tumor necrosis factor
